# Tailored Physiotherapy Combined With Exercises for Enhanced Recovery Post-Below-Knee Amputation in a Diabetic Patient With Peripheral Artery Disease (PAD): A Case Report

**DOI:** 10.7759/cureus.69781

**Published:** 2024-09-20

**Authors:** Simran F Sheikh, Gauri Kariya, Tejaswini Dafe

**Affiliations:** 1 Department of Community Health Physiotherapy, Ravi Nair Physiotherapy College, Datta Meghe Institute of Higher Education and Research, Wardha, IND

**Keywords:** below-knee amputation (bka), exercise, phantom limb pain, postoperative recovery, rehabilitation

## Abstract

This case report summarizes the physiotherapy rehabilitation process for a 59-year-old male patient who had undergone a below-knee amputation due to complications from diabetes mellitus, leading to peripheral artery disease (PAD). In this patient population, early rehabilitation is crucial to achieving maximal outcomes. In the following case report, physiotherapy was started on the second postoperative day following the completion of the below-knee amputation (BKA). A rehabilitation program was designed with consideration of specific challenges presented by his comorbid condition, which emphasized wound care, edema management, and pain relief, followed by early mobilization. His pre-prosthetic training included strengthening and range of motion exercises, exercise training, and techniques to improve posture by using exercises to reduce sensitivity in the residual limb. The phantom pain was well managed, and the patient recovered and experienced effective training in terms of prosthetic fitting. This serves as a testament to the importance of physiotherapy with early and tailored intervention for patients with diabetes and PAD following BKA, which has shown to be efficient in improvising functional and quality of life outcomes through a comprehensive rehabilitation program.

## Introduction

Transtibial amputation or below-knee amputation (BKA) preserves the knee joint while removing the lower leg. This type of amputation is often performed due to conditions that severely impair the lower extremities' blood supply in diseases such as diabetes mellitus (DM), peripheral artery disease (PAD), trauma, or infection. Transtibial amputation is preferred over more proximal amputations because retaining the knee joint significantly improves the potential for mobility and the use of a prosthesis [1]. In 1990, there were approximately 11.37 million cases of traumatic amputation globally. By 2019, this number had increased to approximately 13.23 million cases, showing an increase of 16.4%, and the total number of cases had surged to 552.45 million, an increase of 49.2% [2].

PAD is a cardiovascular condition that restricts blood supply to the legs. The term critical limb ischemia is a distressing form of limb-threatening ischemia. Major symptoms of ischemia are pain at rest and tissue damage such as non-healing ulcers and gangrene [3,4]. DM is a trivial risk factor for lower extremity amputation (LEA). Poorly managed diabetes leads to a range of complications, including muscular, sensory, and ischemic changes, which are significantly threatening and followed by various foot problems and amputation [5].

DM is a chronic disease affecting approximately 19% of the global population. Lower limb amputation is the most common management. It is predicted to increase up to 590 million, according to the Diabetes Federation [6,7]. Elevated diabetes blood glucose levels cause compromising immune function as it will impair the function of white blood cells. The body's function to fight infections and wound healing will be impaired. The most common complication of uncontrolled diabetes is diabetic foot ulcers due to PAD. Diabetic foot ulcers (DFUs) are a common cause of hospitalization in diabetic patients and can progress to the lower extremities [8,9]. DFUs can lead to LEA as they severely affect a patient's quality of life and increase mortality rates due to the impaired wound healing associated with diabetes [10,11].

Physical rehabilitation after a BKA or transtibial amputation plays a crucial role in improving physical function and quality of life. Key interventions include wound management to prevent infection and allow healthy healing of the stump, desensitization techniques such as edema control through compression therapy, and reducing hypersensitivity. Early mobilization is facilitated through the use of assistive devices such as walkers and progressive strength training before prosthetic fitting. Additionally, pain management strategies, including manual therapy, stretching, and mirror therapy, address phantom limb pain and residual limb discomfort, contributing to more successful rehabilitation outcomes. These outcomes include improved ability to use prosthetics, more mobility, greater musculoskeletal endurance, faster walking ability, better fit for the prosthesis, and a higher rate of survival at one year [12,13].

This case report introduces exercises as a novel and effective method for correcting postural deformities. The exercises consist of posture correction techniques aimed at relieving pain and restoring proper body alignment by targeting musculoskeletal imbalances. These exercises are tailored to an individual's specific postural deviations and work to correct compensations resulting from injury, poor habits, or repetitive strain. By encouraging proper alignment, these exercises help minimize joint stress, improve movement efficiency, and prevent chronic pain. Commonly applied for conditions such as back pain, joint discomfort, and posture-related issues, these exercises support long-term musculoskeletal health and overall well-being. The exercises emphasize a holistic approach to rehabilitation by targeting specific muscle groups that are weak or tight, with the goal of restoring proper alignment and function throughout the body. Incorporating these exercises into the rehabilitation process causes a significant change in posture, pain reduction, and better overall body function.

The case report provides a comprehensive overview of a patient's rehabilitation journey following a BKA. It highlights the specific physiotherapy techniques employed, including the implementation of exercises, and discusses the detailed outcomes achieved through this approach. The report outlines how these exercises were integrated into the patient's rehabilitation program, the progress observed in terms of mobility and alignment, and the overall betterment in their life.

## Case presentation

Patient information

A 59-year-old male presented to the hospital with major complaints of an ulcer on his left foot, black discoloration of his toes, and pain in his calf muscles while walking. The patient reported that a wooden stick got plunged into his toes seven days ago. The patient was also diagnosed with diabetes five years ago. Following the patient's examination and investigations, including X-ray and color Doppler, atherosclerotic changes were identified, along with gangrene of the foot that extended to the distal leg. The following day, he underwent transtibial amputation. Additionally, his physiotherapy session commenced on the second postoperative day. The timeline of the patient's admission is presented in Table 1.

**Table 1:** Timeline of the patient's admission

Dates	Events
1/07/24	The patient noticed the changes in his left foot.
10/07/24	The patient was referred to a local hospital and was managed conservatively with medications.
24/07/24	The patient’s symptoms aggravated, so he came to AVBRH hospital.
25/07/24	Patient's investigations were done.
29/07/24	The patient underwent surgery.
31/07/24	The physiotherapy call was started on postoperative day two.

Investigations

**Figure 1:**
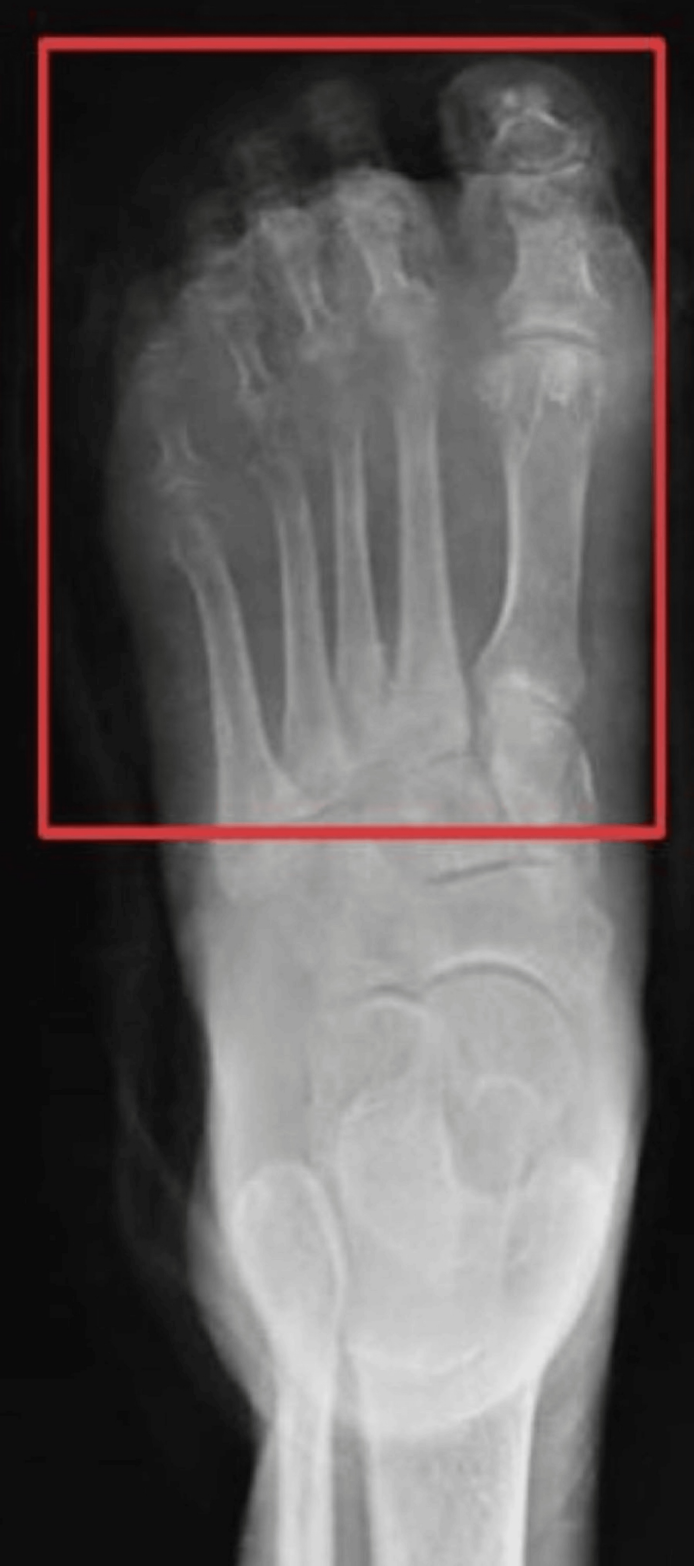
Radiograph of the left foot showing osteophytes

The radiograph revealed multiple osteophytes in proximal phalanges extending to the head of the metacarpals (Figure 1). The color Doppler test showed atherosclerotic changes in the left lower limb.

Clinical findings

**Figure 2:**
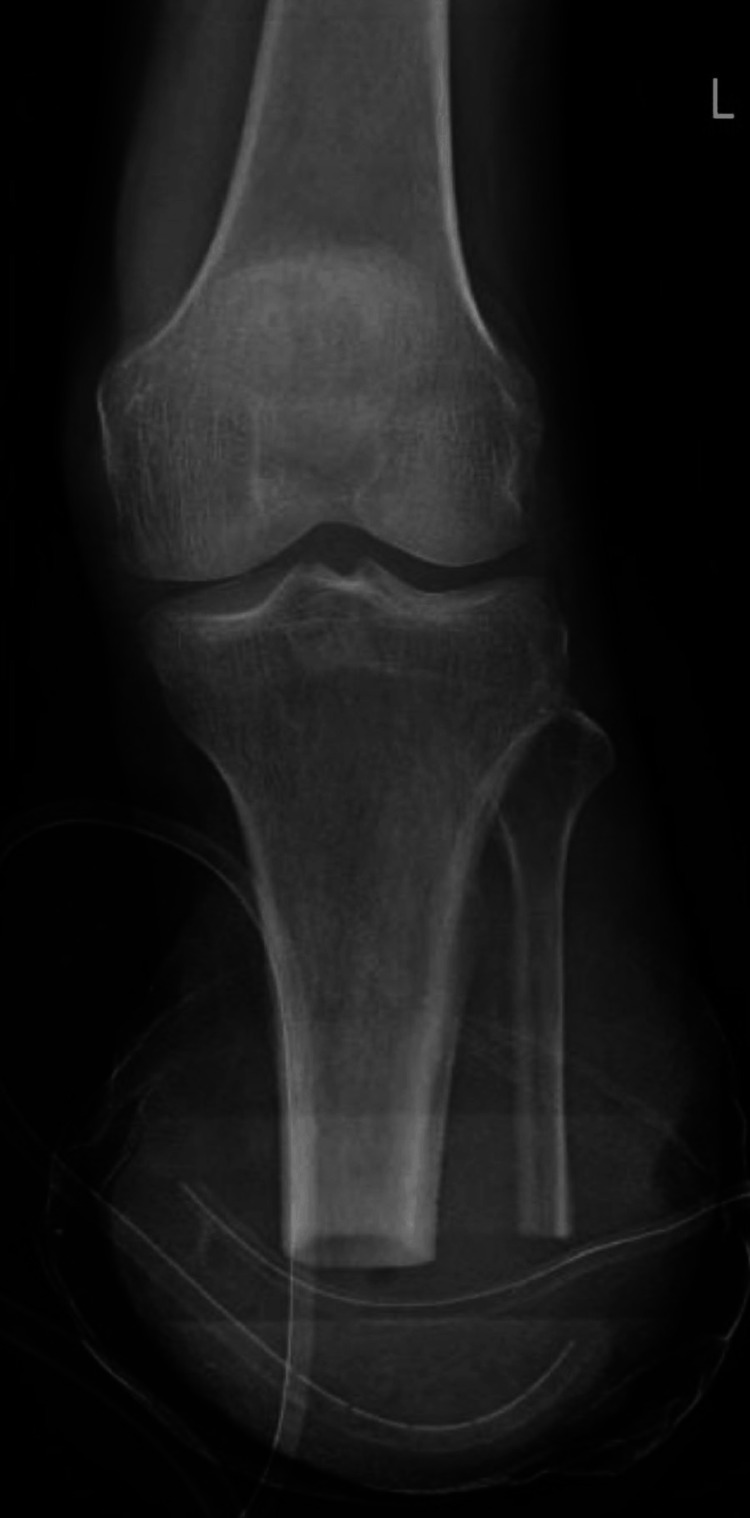
Post-amputation with an inserted drain

A physical examination was conducted on the second postoperative day after obtaining the patient's consent. The patient was alert, cooperative, and fully oriented to time, place, and person. Vital signs indicated hemodynamic stability. The examination was performed with the patient lying supine, the head in a neutral position, and the back supported. The anterior superior iliac spine (ASIS) was aligned, while the leg to the left was observed to be in an abducted and externally rotated position. A rigid dressing was applied to the amputated stump to help prevent swelling and to promote wound healing. A drain was placed in the stump, and no hematoma was observed in the drain tube, indicating no significant postoperative bleeding. Figure 2 shows the radiograph after the transtibial amputation with an inserted drain. The patient reported significant pain in the distal amputated region during movement, with a pain intensity of 9/10 on the numerical pain rating scale (NPRS). The pain was described as pricking in nature, progressive, and aggravated by movement. Grade 3 tenderness was noted on palpation.

**Figure 3:**
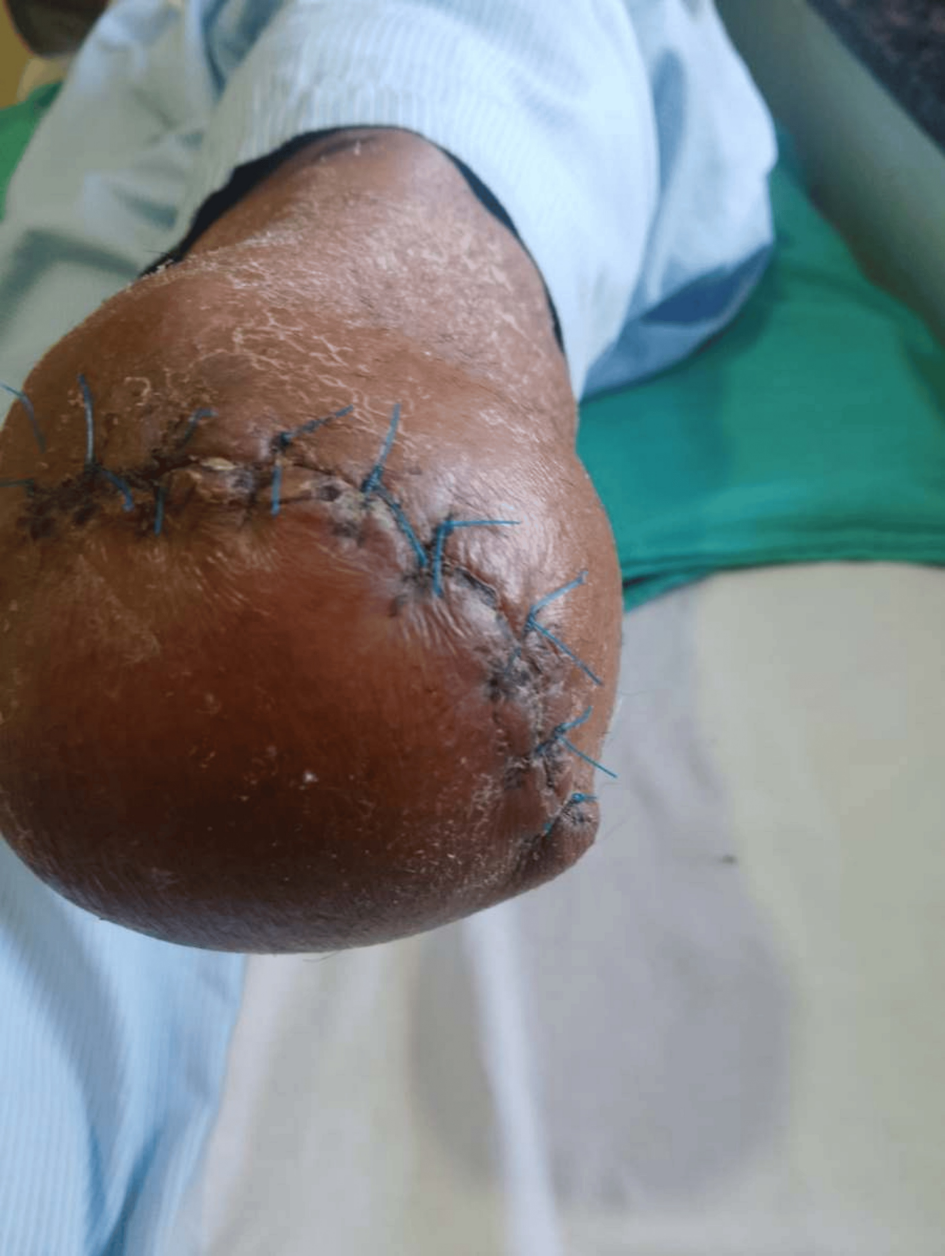
Healthy healing suture

Figure 3 shows a healthy healing suture without discharge. No considerable muscular atrophy was observed, which is positive for early postoperative recovery. Table 2 shows the range of motion, while Table 3 shows the results of manual muscle testing, indicating that the upper limb evaluation demonstrates normal ranges of motion and manual muscle strength.

**Table 2:** Range of motion

Range of Motion	Active Range of Motion		Passive Range of Motion	
	Right	Left	Right	Left
Hip flexion	0-100°	0-50°	0-100°	0-55°
Hip extension	NA	NA	NA	NA
Hip abduction	0-40°	0-20°	0-40°	0-20°
Hip adduction	0-20°	0-10°	0-20°	0-10°
Hip external rotation	0-40°	0-20°	0-40°	0-20°
Hip internal rotation	0-40°	0-20°	0-40°	0-20°
Knee flexion	0-140°	0-80°	0-140°	0-80°
Knee extension	0°	0°	0°	0°
Ankle dorsiflexion	0-30°	Due to amputation, it was unassessable	0-30°	Due to amputation, it was unassessable
Ankle plantarflexion	0-40°	Due to amputation, it was unassessable	0-40°	Due to amputation, it was unassessable

**Table 3:** Manual muscle testing

Manual Muscle Testing	Right	Left
Hip flexors	3/5	2/5
Hip extensors	3/5	2/5
Hip abductors	3/5	2/5
Hip adductors	3/5	2/5
Hip external rotators	3/5	2/5
Hip internal rotators	3/5	2/5
Knee flexors	3/5	2/5
Knee extensors	3/5	2/5
Ankle dorsiflexors	3/5	Due to amputation, it was unassessable
Ankle plantarflexors	3/5	Due to amputation, it was unassessable

Therapeutic intervention

**Figure 4:**
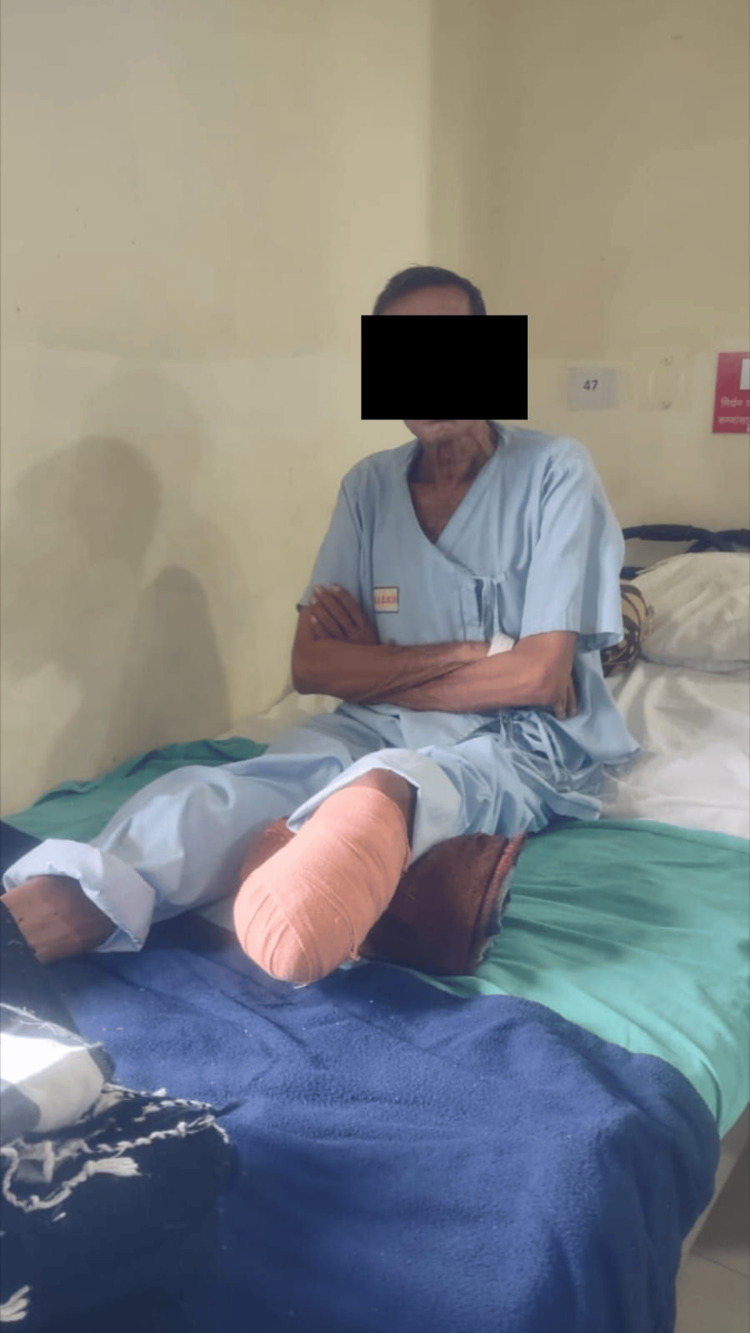
The patient performing a quadriceps set

**Figure 5:**
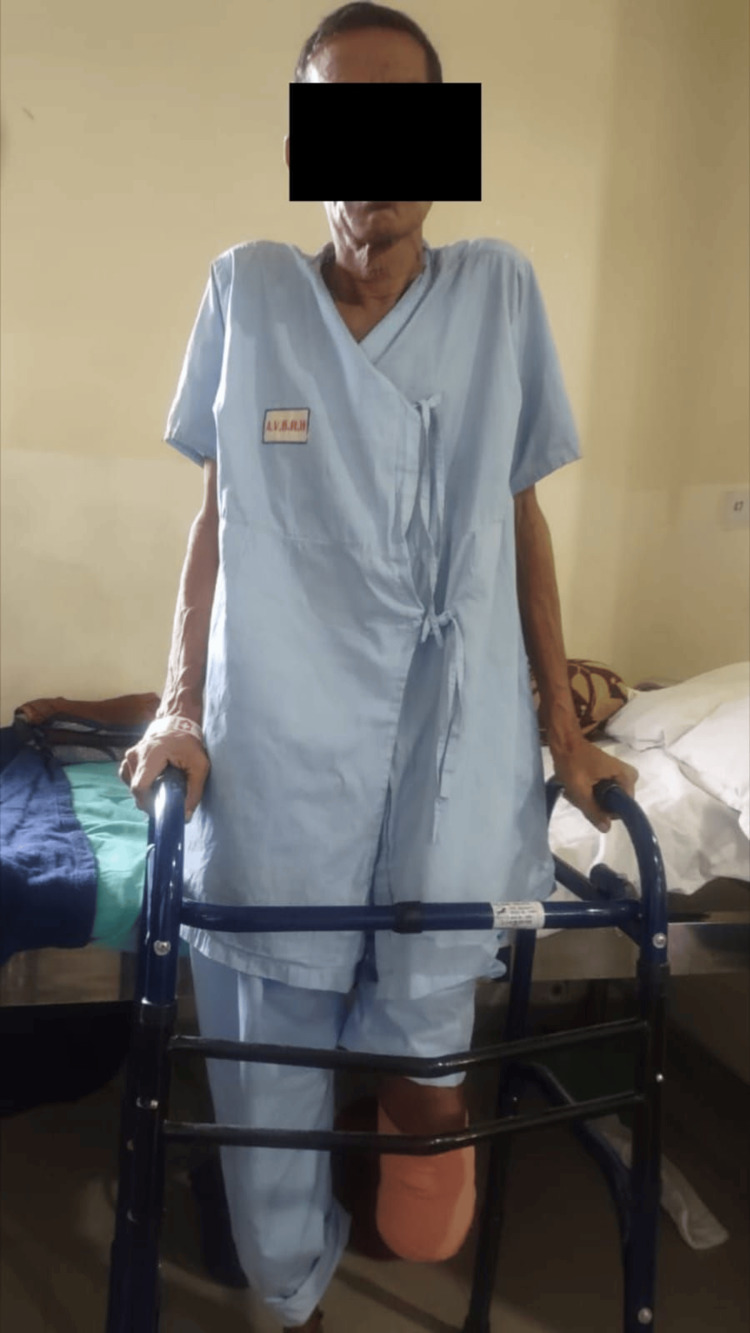
The patient performing partial weight-bearing (PWB) gait training with a walker

Initially, the patient was managed through a surgical procedure involving transtibial amputation. Consequently, following surgical management, postoperative medications and rehabilitation were recommended due to the patient's experience of pain, reduced muscle strength, and decreased functional independence. The key objective of rehabilitation was to help him alleviate the pain, increase his muscular strength, promote wound healing, and subsequently regain his functioning skills and self-reliance. Table 4 summarizes pharmaceutical management, and Table 5 outlines the physiotherapeutic protocol for two consecutive weeks. Figures 4, 5 show the patient performing exercises.

**Table 4:** Pharmaceutical management BD: twice a day; OD: once a day; SOS: if necessary or needed; HS: at bedtime; TDS: three times a day

Medications	Doses	Rationale
Tab Septran	BD	Antibiotic, used to treat bacterial infections
Tab PAN 40 mg	OD	Used to reduce stomach acid
Tab Emeset 4 mg	SOS	Antiemetic, used to prevent nausea and vomiting
Tab Tramadol 50 mg	BD	Used to manage moderate to severe pain
Tab Ecosprin	HS	Antiplatelet, used to prevent blood clotting
Tab Complamina	OD	Vitamin
Tab Gepride	BD	Oral hypoglycemic drug
Diabetes protein powder	TDS	Balance nutrition powder for diabetes

**Table 5:** Physiotherapy management NA: not applicable; ROM: range of motion; μs: microseconds

Goals	Intervention	Procedure	Dosage
Educating patient	To educate the patient and his family on the positive effects of physiotherapy	An essential component of postoperative physical therapy rehabilitation after a transtibial amputation is patient education. Starting on day two, the patient is given an education that helps him manage his goals, participate actively in his recovery, and comprehend his condition, wound care, and mobility exercises	NA
To alleviate pain	1. Transcutaneous electrical nerve stimulation (TENS); 2. Cryotherapy	1. For phantom pain, placement might be on the residual limb or around the stump area, depending on the pain location; 2. An ice pack wrapped in a towel kept around the painful area	1. Utilizes low-intensity, short-duration pulse width of 50-200 μs and high-frequency 50-100 Hz current; 2. Kept for 15-20 minutes for 2-3 times a day
Positioning and edema management	1. Elevation; 2. Avoid prolonged knee flexion; 3. Compression wrapping	1. Keep the affected limb elevated to reduce edema; 2. Encourage lying prone to prevent hip and knee flexion contractures; 3. As the surgical wound is stable, begin gentle compression wrapping with an elastic bandage or shrinker to control swelling and shape the residual limb.	15-20 minutes, 2-3 times daily
To prevent respiratory complications	1. Deep breathing exercises; 2. Incentive spirometry	1. To prevent respiratory complications, especially in patients with reduced mobility; 2. Encourage the use of an incentive spirometer to improve lung function	1 set of 10 repetitions
To prevent muscle atrophy	1. Range of movement exercises (ROM)	Focus on active and passive ROM exercises for the hip and knee to prevent muscle atrophy. Exercises include the hip extension in a prone. Knee extension with a rolled towel under the knee. Gentle hip abduction/adduction. Ankle ROM on the unaffected side	1 set of 10 repetitions
To promote healing of stump and scar management	Infrared radiation therapy	The frequency will depend on the patient's specific needs. Initially, it may be applied daily or several times a week and less frequently as healing progresses	For 10-15 minutes per week for the healing of the stump and 20 minutes in the fourth week for scar management
To strengthen the muscles	1. Strengthening exercises	1. Isometric exercises: *Quadriceps sets (press the knee into the bed). *Gluteal sets (squeeze the buttocks); 2. Core strengthening: Pelvic tilts and abdominal bracing; 3. Upper limb strengthening	1 set of 10 repetitions
Bed mobility and transfers	1. Rolling; 2. Transfers	1. Rolling from supine to side lying and then to sitting; 2. Safe transfer techniques from bed to wheelchair using assistance as needed.	NA
Balance and coordination	1. Seated Balance Exercises; 2. Weight Shifting	1. Practice sitting balance with and without upper limb support; 2. Gentle weight-shifting exercises while seated or standing (with support)	NA
Mobility training	1. Gait training with the assistance of a walker	1. Begin standing exercises using a walker, focusing on maintaining a neutral pelvis and knee extension. 2. Non-weight-bearing (NWB) or partial weight-bearing (PWB) gait training with walker	For 10-15 minutes. Started in the second week
Posture correction	Exercises	1. Static back; 2. Seated forward bend; 3. Arm circles; 4. Hip crossover stretch	1 set of 10 repetitions
Pre-prosthetic teaching	1. Pre-prosthetic gait training; 2. Prosthetic preparation	1. Weight distribution and limb care; 2. Compression bandaging and massaging. Soft tissue mobilization	NA

Follow-up and outcome

Following the physiotherapy rehabilitation, the patient experienced no pain and an improvement in range of motion. He was able to walk with a walker and perform some activities. The patient reported improved muscle strength and range of motion. Table 6 shows the range of motion, while Table 7 presents the results of manual muscle testing. Table 8 shows the NPRS and Beck depression inventory scale scoring, taken on day two and fourth week as outcome measures.

**Table 6:** Pre- and post-intervention manual muscle testing

Manual Muscle Testing	Pre-intervention		Post-intervention	
	Right	Left (affected)	Right	Left (affected)
Hip flexors	3/5	2/5	4/5	3/5
Hip extensors	3/5	2/5	4/5	3/5
Hip abductors	3/5	2/5	4/5	3/5
Hip adductors	3/5	2/5	4/5	3/5
Hip external rotators	3/5	2/5	4/5	3/5
Hip internal rotators	3/5	2/5	4/5	3/5
Knee flexors	3/5	2/5	4/5	3/5
Knee extensors	3/5	2/5	4/5	3/5
Ankle dorsiflexors	3/5	Due to amputation, it was unassessable	4/5	Due to amputation, it was unassessable
Ankle plantarflexors	3/5	Due to amputation, it was unassessable	4/5	Due to amputation, it was unassessable

**Table 7:** Pre- and post-interventional range of motion

Range of Motion	Pre-interventional		Post-interventional	
	Right	Left (affected)	Right	Left (affected)
Hip flexion	0-100°	0-50°	0-100°	0-55°
Hip extension	NA	NA	0-30°	0-30°
Hip abduction	0-40°	0-20°	0-40°	0-40°
Hip adduction	0-20°	0-10°	0-20°	0-20°
Hip external rotation	0-40°	0-20°	0-40°	0-40°
Hip internal rotation	0-40°	0-20°	0-40°	0-40°
Knee flexion	0-140°	0-80°	0-140°	0-120°
Knee extension	0°	0°	0°	0°
Ankle dorsiflexion	0-30°	Due to amputation, it was unassessable	0-30°	Due to amputation, it was unassessable
Ankle plantarflexion	0-40°	Due to amputation, it was unassessable	0-40°	Due to amputation, it was unassessable

**Table 8:** Outcome measures NPRS: numerical pain rating scale

Outcome Measure	Pre-intervention	Post-intervention
NPRS	9/10	2/10
Beck depression inventory scale	31/63	9/63

## Discussion

The World Health Organization (WHO) conducted a survey that concluded that approximately 40 million people in developing countries underwent amputation surgery. In India, the 2011 census revealed that 2.68 crore individuals, or approximately 2.22% of residents, have disabilities, with amputation being a significant reason. Despite this, only about 5% of those in need have access to prosthetic devices [14,15]. This case underscores the critical need for a multidisciplinary approach in the rehabilitation of below-knee amputees.

Physiotherapy is essential in overcoming physical challenges, optimizing the use of prosthetics, and ensuring long-term functional success. The key components of successful rehabilitation include early intervention, patient education, and continuous support. Physiotherapy initiated in the acute postoperative phase focuses on preventing contractures, reducing edema, and improving mobility for bedside activities and transfers. Proper wound care, including regular dressing changes and edema control, is essential to promote healing and prevent infection. Compression techniques, such as elastic bandaging or stump shrinkers, help reduce swelling and shape the residual limb for prosthesis fitting​. After surgical removal of the limb, an elastic compression bandage is applied, which shapes the stump and assists in wound healing. Early mobility is encouraged after teaching and assisting in wheelchair transfers and progressing to the walker. Gait training is essential for learning to walk with a prosthetic limb, and balance exercises help restore coordination and postural control. Pain management techniques, including manual therapy and the use of modalities such as TENS (transcutaneous electrical nerve stimulation), are also implemented to address phantom limb pain and discomfort in the residual limb.

After the stump has been healed, prosthesis facilitation is started. In this phase, patients are encouraged to strengthen proximal muscles and retrain gait [16-18]. The exercise physiology focuses on restoring proper alignment and function of the body through a series of specific exercises and postural corrections. This approach aims to address musculoskeletal imbalances and reduce pain by improving alignment and correcting dysfunctional movement patterns. The exercises are designed to stretch and strengthen key muscle groups, promote joint stability, and enhance overall body mechanics, ultimately leading to better posture, reduced pain, and improved function. When these exercises are combined with TENS, these exercises reduce pain and support postural correction. TENS stimulates nerves, disrupts pain signals from the brain, and helps reduce pain, while these exercises help correct musculoskeletal imbalances and posture correction [19].

Balance and posture training are essential for amputees to regain their independence following amputation. Numerous studies have explored innovative approaches to enhance rehabilitation in these cases. One such study, conducted in 2012, investigated the use of video games for balance training in children and adolescents with lower limb amputations. The findings were promising, demonstrating potential long-term benefits for rehabilitation [15,20,21].

## Conclusions

This case report underscores the increasing global prevalence of traumatic amputations and highlights the essential role of rehabilitation in enhancing outcomes for individuals who have undergone BKA. We discuss the case of a 59-year-old male whose physiotherapy rehabilitation commenced following his transtibial amputation due to diabetes-related PAD leading to gangrene in his foot. After surgery, the rehabilitation process focused on pain management, wound healing, muscle strengthening, and restoring mobility. The following case illustrates the benefits of standardized physiotherapy protocol when combined with postural correction exercises. Effective strategies were employed to control postsurgical pain, which is crucial for enabling active participation in rehabilitation exercises and overall recovery. Close monitoring and interventions were implemented to promote proper healing of the surgical site, reducing the risk of infection and other complications. The case emphasizes the effectiveness of a standardized physiotherapy protocol that integrates these elements.

Furthermore, it highlights the added benefits of incorporating postural correction exercises into the rehabilitation plan. These exercises are designed to address postural imbalances and musculoskeletal dysfunctions by promoting proper alignment and movement patterns. By focusing on realigning the body and correcting dysfunctional postures, these exercises can complement the core physiotherapy interventions, leading to improved functional outcomes and overall recovery. This detailed approach not only underscores the importance of a structured rehabilitation protocol but also demonstrates how integrating innovative methods including exercises can further enhance the rehabilitation process and contribute to a more comprehensive and effective recovery strategy for patients undergoing BKA.
